# Psychological health during the coronavirus disease 2019 pandemic outbreak

**DOI:** 10.1177/0020764020925835

**Published:** 2020-05-21

**Authors:** Sonia Mukhtar

**Affiliations:** University of Management and Technology, Lahore, Pakistan

**Keywords:** COVID-19, coronavirus pandemic, mental health, social and behavioral epidemiology, psychological problems, psychosocial issues, misinfodemics, stigma, resilience, coping, mindfulness and well-being

## Abstract

**Background:**

The current ongoing pandemic outbreak of COVID-19 (Coronavirus Disease 2019) has globally affected 213 countries and territories with more than 2.5 million confirmed cases and thousands of casualties. The unpredictable and uncertain COVID-19 outbreak has the potential of adversely affecting the psychological health on individual and community level. Currently all efforts are focused on the understanding of epidemiology, clinical features, mode of transmission, counteract the spread of the virus, and challenges of global health, while crucially significant mental health has been overlooked in this endeavor.

**Method:**

This review is to evaluate past outbreaks to understand the extent of adverse effects on psychological health, psychological crisis intervention, and mental health management plans. Published previous and current articles on PubMed, EMBASE, Google Scholar, and Elsevier about psychological impact of infectious diseases outbreaks and COVID-19 has been considered and reviewed.

**Comments:**

COVID-19 is leading to intense psychosocial issues and comprising mental health marking a secondary health concern all around the world. Globally implementing preventive and controlling measures, and cultivating coping and resilience are challenging factors; modified lifestyle (lockdown curfew, self-isolation, social distancing and quarantine); conspiracy theories, misinformation and disinformation about the origin, scale, signs, symptoms, transmission, prevention and treatment; global socioeconomic crisis; travel restrictions; workplace hazard control; postponement and cancellation of religious, sports, cultural and entertainment events; panic buying and hoarding; incidents of racism, xenophobia, discrimination, stigma, psychological pressure of productivity, marginalization and violence; overwhelmed medical centers and health organizations, and general impact on education, politics, socioeconomic, culture, environment and climate – are some of the risk factors to aggravate further problems.

## Introduction

The ongoing pandemic COVID-19 (*Co*rona*vi*rus *D*isease 20*19*) has become a threat to psychological health as previous research works revealed profound and wide range of psychosocial impact on individual, community and international levels during past outbreaks of infectious diseases (Xiang, 2020). During previous outbreaks, the psychological impact on non-infected community revealed significant psychiatric morbidities, negative emotions, and poor psychosocial and coping responses toward the outbreak of infectious diseases and consistent worry about contracting the disease (Van Bortel, 2016). Currently, there is a paucity of information on the psychological impact of the general public, confirmed and suspected cases, medical staff and law enforcement agents during the outbreak of COVID-19 pandemic, especially in the context of mental health impact. This has become even more pertinent given the uncertainty and unpredictability revolving around the outbreak of coronavirus pandemic of such unparalleled magnitude and intensity. Conspiracy theories, false claims, misinformation and disinformation (mainly exclaiming coronavirus as Unbreakable, Unstoppable, Unbeatable) are only exacerbating the mental composure of general public. Many of the research works related to the COVID-19 outbreak focus on identifying the epidemiology, clinical characteristics, genomic characterization of the virus, clinical features, data on mode of transmission and its route, reservoirs, incubation period, symptoms and clinical outcomes, including survival and mortality rates; counteracting the spread of the virus; and management of global health governance (Chen, 2020; Corman et al., 2020; Huang, 2020; Lu, 2020; Mukhtar, 2020). The compelling emergency is calling for the comprehensive research work on psychological health and mental well-being of the community in the face of COVID-19.

There was an observed neuropsychiatric linkage between SARS (severe acute respiratory syndrome) and mental health problems with severe psychiatric comorbidities like depression, panic attacks, anxiety attacks, psychomotor excitement, suicidal deaths, delirium and psychotic symptoms (Xiang et al., 2020). Also the lives affected by COVID-19 are at further stake due to the perpetuated potential adverse effects. For instance, during travel restrictions and postponement and cancelation of religious, sports, cultural and entertainment events, people in quarantine may experience anger, loneliness, boredom and anxiety, and symptoms of cough, fever, myalgia and fatigue may cause emotional distress and feelings of fear of contracting COVID-19 (Xiang et al., 2020). While scientists, clinicians, local and international health organizations and authorities, epidemiologists and virologists are working on many unanswered questions of this novel outbreak, general public, global media and opinion-makers are responding to this uncertainty based on a limited confirmed/unconfirmed knowledge. This has further elevated the repercussions in the lives of people in the wake of COVID-19 and calls for the new database of research on psychological health. Nevertheless, the impact of transmitted viral infection on psychological health has not been acknowledged in its entirety, which challenges the patients and the general population.

Literature search in this commentary was carried out on PubMed, EMBASE, Google Scholar and Elsevier with keywords ‘COVID-19’, ‘coronavirus pandemic’, ‘Pakistan’ and ‘mental health’, ‘psychological impact’, ‘psychosocial issue’ and ‘distress’. Researches were further reviewed and screened for the relevance of literature review. The current commentary paper focused on the present COVID-19 pandemic mental health concerns and psychological interventions for affected individuals, at-risk population and health care staff.

## Commentary

Health organizations and health care professionals are focusing on controlling the COVID-19 pandemic by recommending self-isolation, social distancing and quarantine, with the slightest emphasis on the impact of psychological health (‘Coronavirus disease situation reports’, 2020). The emergence, prevalence and transmission of COVID-19 are beyond physical health, and emotional distress, anxiety, fear, depression, suicidality, public stigma, discrimination, racism, xenophobia, posttraumatic symptoms and sleep disturbance are some of the consequences on psychological health. The physical counteractive measures toward managing COVID-19 include early identification and separation of suspected cases, biological and clinical data collection, consensus of expert medical interventions, establishment of quarantine units and strengthening of medical staff in the affected regions (Ford-Jones & Chaufen, 2017; ‘Report of the WHO’, 2020; Severance et al., 2011). These infection prevention and control practices interventions have proven to be effective for combating the pandemic but have serious psychological health impacts on the medical teams and the general public (Rubin & Wessely, 2020).

## Preparedness and proactive infection control

The objective of health care authorities across the world is to ensure preparedness and proactive infection control by the health care system and provide recommendations for the benefit of public health. Readiness is measured in terms of availability of resources, emergency funds and guidance manuals for public information about COVID-19 and control measures; collaborative working of public health departments and pharmaceutical companies; availability of medical and personal protective equipment; and established health care units on prevention, management and control of the pandemic – but with little attention on mental health.

## Mental health impact on patients, health care staff, law enforcement agents and general public

Past studies conducted to assess the psychological and immediate stress outcomes on patients who were quarantined during Middle East respiratory syndrome (MERS) showed higher impact events score on sleep, numbness, anxiety and depression, and the results of psychological distress were consistent with affected individuals in Nigeria on the impact of Ebola virus (Lee et al., 2018; Mohammed et al., 2015). Other studies detailing the impact of psychological trauma of bereavement in the case of MERS stated that surviving individuals were stigmatized, marginalized and socially isolated even after successful treatment (Shigemura et al., 2020; Sim, 2016). Extended period of incubation longer than usual due to public uncertainty is the byproduct of infiltrated misinformation and disinformation on social media. The isolated state of COVID-19 holds similarity with MERS and SARS as similar claims are circulating on social media about the uncertainty and unpredictability around COVID-19, instigating fear, anxiety, panic and worries in the general public. Other studies (Batawi et al., 2019; Cheung et al., 2008) on psychiatric impact of SARS survivors revealed the intense post-traumatic stress disorder (PTSD) symptoms and exacerbated depressive symptoms. This correlated to the suicide deaths of older adults and low quality of life among affected individuals and unaffected general public, respectively (Mak et al., 2009).

Previous studies on the infectious outbreaks of SARS, MERS and Ebola revealed the severity of emotional distress not only in the general public but also among many medical practitioners and law enforcement agents who faced PTSD, depression, anxiety, exhaustion and burnout at the onset, during and even after the outbreak of such epidemics (Lee et al., 2018). The case of COVID-19 poses more significant mental health deterioration since medical practitioners and nurses are equally susceptible to the infectious transmitting disease due to inadequate personal protective equipment (PPE), burnout, exhaustion, frustration, hopelessness, discrimination, isolation, patients with negative emotions and lack of contact with their families (Kang et al., 2020).

This worldwide public health concerns the role and responsibility of medical workers, global impact of infection, impact of economic activities on travel and trade restrictions, and equitable care of public welfare and individual rights during the spread of pandemics. Psychological health effects could be minimized by avoiding excessive exposure to COVID-19 media coverage (a prevalent national pastime, especially binge-watching news channels), maintaining positive lifestyle and compassionately consoling others as well. Resilience training programs for medical staff, law enforcement agents and general public to cope with the aftereffects of the pandemic of this severity and intensity should be introduced: (a) family–work life balance; (b) reliable, authentic and timely incorporated information about the infectious disease and its consequences on psychological health; (c) educating and preparing communities for pandemics and epidemics in the future; and (d) and validating and valuing frontline’s staff’s contribution.

## Maintaining resilience, coping, mindfulness and well-being

‘Men are disturbed not by things, but by the view which they take of them’ is the philosophical origin of the active, directive, structured and time-framed intervention approach to address, mitigate, assess, treat and manage the plethora of psychological, mental, emotional, behavioral and social and even the interplay of bio-psycho-socio-spiritual domain at the expense of COVID-19. Mental health services, facilities and specialized psychiatric treatment teams including psychologists, psychiatrists and psychiatric nurses should be established to address psychological health concerns in the general public. Individuals and communities could deliberately cultivate resilience, healthy coping strategies, mindfulness and well-being. The potential for resilience, coping, mindfulness and well-being are neither unique traits that one possess (or not) nor outcomes of absence of posttraumatic stress. The capacity for resilience is a process of adaptation. Coping mechanism is a learned pattern of behavior which one develops over the period of time. Mindfulness is the psychological process of purposely bringing one’s attention in the present moment which one develops with practice. Well-being is the experience of being comfortable in their situation. These all are processes and they can be acquired with practice. Resilience, coping, mindfulness and well-being are not single dichotomous outcomes measured – strengthening these processes deliberately reinforced practice by experiencing and learning dynamically.

## Blocking out (*mis*)infodemics, misinformation and disinformation

Empirical and scientific information about the COVID-19 prevalence, prevention, controlling and treatment plan, progress report and updated status of health manuals (in native languages) should be disseminated to frontline medical teams and law enforcement agents, patients, caregivers and families, and general public. Media accumulates conspiracy theories, misinformation and disinformation about the origin, scale, signs, symptoms, transmission, prevention and treatment; media exposure accumulates ever-emerging threats, and repeated exposure to these events increases the symptoms of distress. Conspiracy theories, false claims, misinformation and disinformation (mainly exclaiming coronavirus as Unbreakable, Unstoppable, Unbeatable) are only exacerbating the mental composure of general public. The authorized health organizations and state should enforce and ensure reliable online information through reliable sharing platforms to provide and promote tele-psychological counseling and psychotherapeutic treatments to reduce the impact of psychological health during COVID-19.

## COVID-19, stigma and psychological pressure of productivity

There is a percolating impression among the general public regarding the lockdown as a large group of people assumed it as holidays or vacations and coerced others for optimal functioning in utilizing their time and forcibly engaging in occupational or academic activities. Circulating social media quotes such as ‘If you don’t come out of this quarantine with a new skill, your side-hustle started, or more knowledge gained . . . then you never lacked time, you lacked discipline’ are further compromising the mental health of individuals and society. Messages such as ‘if you don’t come out of this quarantine with a new skill, your side-hustle started, or more knowledge gained, then you are doing just fine’ should be circulated since not everyone can perceive a traumatic event as an opportunity of learning. This stigmatizing psychological pressure has further aggravated feelings of guilt, shame, regret, sadness, self-pity, anger, internalized emotions, overwhelmed feelings, negative self-talk, unrealistic expectations and perceived sense of failure. The psychological pressure of competing in collecting maximum tasks than other peers, harnessing herd of followers and subscribers, producing occupational and academic outcomes under the coercion of work or family, and downplaying the trauma in own self and others will have devastating effects on mental well-being (Mukhtar, 2020).

## COVID-19 and vulnerable population

Lockdown resulting in self-isolation, quarantine and social distancing is far beyond than leisure time vacations for improved functioning – it is a collective traumatic event which poses serious threat to people and have resulted in great loss of lives and property for every individual (Mukhtar, 2020). COVID-19 is an individual and collective traumatic event and directly or indirectly has affected every individual in the world. All efforts should be directed toward minimizing the negative effects of this traumatic COVID-19 pandemic event on ‘survivors’. Vulnerable population such as children, older adults, pregnant women, people with existing physical and mental illnesses, victims of abuse and violence, living with abusers and perpetrators, people living below the poverty line and other individuals are susceptible of not just contracting the coronavirus but the psychological trauma as well. Many people are going through interpersonal traumatic events as well in addition to the collective traumatic COVID-19: domestic violence (gender-based violence), abuse, financial burden, loneliness, emotional and behavioral problems, grief and bereavement, fear of losing family, mental health issues, and physical injuries or fatalities.

## Psychological crisis intervention and psychological first aid

Psychological crisis intervention (PCI) and psychological first aid (PFA) are the early interventions that focus on the psychological health of the affected individuals and offer a designed tool by providing psychosocial support to mitigate distress during outbreaks such as COVID-19. PCI and PFA are essential for emergency management to orient emotionally overwhelmed survivors through practical help, contact, engaging, safety and comfort, and through addressing stress-related reactions. PFA model (Everly et al., 2012) consists of developing rapport through active and empathetic reflective listening, assessment and evaluation of psychological needs, prioritization depending on the severity of emergent cases, cognitive and behavioral interventions to mitigate distress, and disposition and follow-up until stabilization of the situation through constant support and regular monitoring.

## Potential strategies

Although impact of this pandemic on global psychological health is not yet registered and measured, similar information from previous research works could offer an explanation and insight. Early and timely psychiatric interventions should be delivered by mental health practitioners to cope with the outbreak of high-mortality infectious diseases (Mukhtar, 2020; Shantanu & Kearsley, 2020). The current pandemic COVID-19 is causing devastating psychosocial health concerns such as stress, distress, fear, anxiety, depressive symptoms, sleep disturbances, denial, anger, frustration and mistrust in the general public (Mukhtar, 2020; Rana et al., 2020). For medical staff, these psychological problems are related to attention and decision-making capacities which could hamper the fight against COVID-19. The prevalence of psychological problems in the general population has been ranging from 4% to 41% of posttraumatic symptoms and 7% of depressive symptoms (Kang et al., 2020). During any community crisis, people seek out event-related information to attain the illusion of control to exude the fear of the unknown which leads to higher anxiety, and in the case of misleading misinformation and disinformation on social media, distorted perception of risk, extreme fear of unknown/uncertainty and public panic may lead to stigmatization, marginalization and scapegoats (Mowbray, 2020; Mukhtar, 2020). And although studies on COVID-19 are scarce, several authors have predicted the possible repercussions on psychological and physiological health not only on the vulnerable but also on the general population (Kang et al., 2020). Psychological interventions and psychosocial support would improve the public mental health during the outbreak of pandemic COVID-19.

## Conclusion

Substantial evidence from the past studies of epidemics on the impact of psychological health has shown psychosocial consequences in the affected individuals and in the general population. The emerging global mental health issues relative to COVID-19 pandemic may evolve into long-lasting health problems permeated through feelings of vulnerability, isolation/quarantine, fear, anxiety, psychological distress, psychosocial stressors, posttraumatic symptoms, stigma and xenophobia. It is vital to emphasize the psychological health and well-being (physical, economic, social, mental, emotional, psychological, spiritual, development and engaging activity, quality of life, life satisfaction and domain-specific satisfaction) of the population through proactive psychological interventions during the COVID-19 pandemic.
